# Therapeutic potential of TAS-115 via c-MET and PDGFRα signal inhibition for synovial sarcoma

**DOI:** 10.1186/s12885-017-3324-3

**Published:** 2017-05-16

**Authors:** Shutaro Yamada, Yoshinori Imura, Takaaki Nakai, Sho Nakai, Naohiro Yasuda, Keiko Kaneko, Hidetatsu Outani, Satoshi Takenaka, Kenichiro Hamada, Akira Myoui, Nobuhito Araki, Takafumi Ueda, Kazuyuki Itoh, Hideki Yoshikawa, Norifumi Naka

**Affiliations:** 1 Department of Orthopaedic Surgery, Osaka University Graduate School of Medicine, 2-2 Yamadaoka, Suita, Osaka, 565-0871 Japan; 2 Musculoskeletal Oncology Service, Osaka International Cancer Institute, 3-1-69 Otemae, Chuo-ku, Osaka, 541-8567 Japan; 3 Department of Orthopaedic Surgery, Osaka National Hospital, 2-1-14 Hoenzaka, Chuo-ku, Osaka, 540-0006 Japan; 4 Research Institute, Nozaki Tokushukai, 2-10-50 Tanigawa, Daitou, Osaka, 574-0074 Japan

**Keywords:** TAS-115, Synovial sarcoma, C-MET, PDGFRα, Molecular targeted therapy

## Abstract

**Background:**

The prognosis of synovial sarcoma (SS), an aggressive soft tissue sarcoma, remains poor. We previously reported that c-MET or platelet-derived growth factor receptor α (PDGFRα) signalling pathway is related to SS progression based upon the findings of phospho-receptor tyrosine kinase (RTK) arrays. TAS-115 is a novel c-MET/ vascular endothelial growth factor receptor-targeting tyrosine kinase inhibitor that has been shown to inhibit multiple RTKs. Here we aimed to investigate the therapeutic potential of TAS-115 against SS.

**Methods:**

We first evaluated which signalling pathway was relevant to the viability of three human SS cell lines: Yamato-SS, SYO-1 and HS-SY-II. Next, we assessed the anticancer activity and mechanism of action of TAS-115 in these SS cell lines. Finally, we compared the ability of TAS-115 to inhibit c-MET and PDGFRα phosphorylation with that of pazopanib.

**Results:**

We classified the SS cell lines as c-MET-dependent or PDGFRα-dependent based upon the differences in the signalling pathway relevant for growth and/or survival. We also found that c-MET and PDGFRα were the primary activators of both phosphatidylinositol 3-kinase/AKT and mitogen-activated protein kinase pathways in c-MET-dependent and PDGFRα-dependent SS cells, respectively. TAS-115 treatment blocked the phosphorylation of PDGFRα as well as that of c-MET and their downstream effectors, leading to marked growth inhibition in both types of SS cell lines in in vitro and in vivo studies. Furthermore, PDGFRα phosphorylation, on at least four representative autophosphorylation sites, was impeded by TAS-115 equivalently to pazopanib.

**Conclusions:**

These experimental results have demonstrated the significance of c-MET and PDGFRα signalling for growth and/or survival of SS tumours. TAS-115 monotherapy may benefit SS patients whose tumours are dependent upon either c-MET or PDGFRα signalling by functioning as a multiple tyrosine kinase inhibitor to suppress c-MET as well as PDGFRα pathways.

**Electronic supplementary material:**

The online version of this article (doi:10.1186/s12885-017-3324-3) contains supplementary material, which is available to authorized users.

## Background

Synovial sarcoma (SS) is a malignant soft tissue sarcoma characterized by a recurrent chromosomal translocation [t(X;18)(p11;q11)] that forms the fusion protein, SS18-SSX [1]. SS accounts for 5%–10% of all soft tissue sarcomas and mainly affects adolescents and young adults [2]. This disease commonly presents in the extremities (80%) [2] and distant metastases tend to be found mainly in the lungs [3]. Although the mainstay of treatment comprises wide surgical excision, chemotherapy and radiotherapy, the 5-year overall survival rate of SS is only 30%–70% [3–7]. Therefore, developing novel therapeutic approaches for SS is urgently needed.

We previously reported that c-MET or platelet-derived growth factor receptor α (PDGFRα) signalling pathway was relevant for SS progression, based upon the findings of phospho-receptor tyrosine kinase (RTK) arrays [8]. c-MET, an RTK encoded by the *c-met* proto-oncogene, is known to be a hepatocyte growth factor (HGF) receptor [9]. Activation of the HGF/c-MET axis in cancer has been reported to be involved in cellular proliferation, survival, migration and angiogenesis [10]. We have found that a selective c-MET inhibitor suppresses the growth of Yamato-SS cells, but fails to suppress the growth of SYO-1 or HS-SY-II cells [11]. PDGFRα and PGDFRβ signalling indirectly promotes tumour development by activating the mesenchymal cells in the tumour microenvironment and directly stimulates the growth of malignant cells [12]. Pazopanib, a PDGFR/ vascular endothelial growth factor receptor (VEGFR)/ c-kit (stem cell factor receptor) inhibitor [13], is the only tyrosine kinase inhibitor approved for advanced soft tissue sarcomas in Japan. Hosaka et al. showed that pazopanib suppressed the growth of SYO-1 and HS-SY-II cells through inhibition of the PDGFRα and phosphatidylinositol 3-kinase (PI3K)/AKT pathways [14]. Based upon these studies, we hypothesize that inhibition of the c-MET or PDGFRα signalling pathway would be a therapeutic strategy for the treatment of SS.

TAS-115, a novel c-MET/VEGFR-targeting tyrosine kinase inhibitor that exerts its effect via ATP antagonism, has been reported to inhibit multiple RTKs [15]. Recently, it was reported that TAS-115 had a favourable tolerability profile and exhibited antitumour activity in human gastric cancer [15, 16] and in human lung cancer [17, 18] via inhibition of c-MET/VEGFR signalling. However, the efficacy of this drug for soft tissue sarcomas remains unclear.

In the present study, we first evaluated the phosphorylation status of RTKs in three human SS cell lines, Yamato-SS, SYO-1 and HS-SY-II, and then investigated which RTK was critical for the viability of each of these cell lines. Next, we tested the antitumour activity and the mechanism of action of TAS-115 in these SS cells. Finally, we compared the inhibitory activity of TAS-115 on the c-MET and PDGFRα pathways with that of pazopanib. On the basis of our observations, we discuss the potential clinical value of TAS-115 monotherapy, via c-MET and PDGFRα signal inhibition, in patients with SS.

## Methods

### Cell lines

The Yamato-SS cell line was established from surgically resected tumours in our laboratory, as previously described [19]. SYO-1 was kindly supplied by Dr. Ozaki (Okayama University, Okayama, Japan) [20]. HS-SY-II [21] was provided by the RIKEN BRC (Tsukuba, Japan) through the National Bio-Resource Project of the MEXT, Japan. We authenticated Yamato-SS and HS-SY-II through short tandem repeat inspection. SYO-1 was confirmed by the expression of the *SS18-SSX2* fusion gene by reverse transcription polymerase chain reaction. Yamato-SS and SYO-1 cells originally derived from biphasic synovial sarcomas, while HS-SY-II originated from a monophasic synovial sarcoma [19–21]. These cells were cultured in Dulbecco’s Modified Eagle’s Medium (Life Technologies, Carlsbad, CA, USA) containing 10% foetal bovine serum (FBS; Sigma-Aldrich, St. Louis, MO, USA) at 37 °C with 5% CO_2_ and 100% humidity.

### Reagents and antibodies

TAS-115 [4-[2-fluoro-4-[[[(2-phenylacetyl)amino]thioxomethyl]amino]-phenoxy]-7-methoxy-N-methyl-6-quinolinecarboxamide] and pazopanib [5-[[4-[(2,3-dimethyl-2H-indazol-6-yl)methylamino]-2-pyrimidinyl]amino]-2-methylbenzenesulfonamide] were provided by Taiho Pharmaceutical Co., Ltd. (Tsukuba, Japan) and Novartis Pharma AG (Basel, Switzerland), respectively. According to the manufacturer’s instructions, TAS-115 and pazopanib were suspended in dimethyl sulfoxide (DMSO, Sigma-Aldrich) for in vitro experiments. TAS-115 and pazopanib were diluted to the appropriate concentrations for in vivo experiments, according to the manufacturer’s instruction. Recombinant human (rh) PDGF-BB was obtained from Sigma-Aldrich.

Antibodies against PDGFRα (#7074), p-PDGFRα (Tyr^754^; #2992, Tyr^849^; #3170, Tyr^1018^; #4547), c-MET (#8198), p-MET (Tyr^1234/1235^; #3077), AKT (#4691), p-AKT (Ser^473^; #4060), ERK (#4695), p-ERK (Thr^202^/Tyr^204^; #4370), PARP (#9542) and β-actin (#4970) were purchased from Cell Signaling Technology, Inc. (Danvers, MA, USA). All of these antibodies were used at 1:1000 dilution for immunoblot analyses. An antibody against p-PDGFRα (Tyr^762^; AF21141) was purchased from R&D systems (Minneapolis, MN, USA). This antibody was used at a concentration of 0.5 μg/ml for immunoblot analyses. An antibody against PCNA (sc-56) was purchased from Santa Cruz Biotechnology, Inc. (Dallas, TX, USA) and used at a concentration of 1:50 for immunohistochemistry. Horseradish peroxidase (HRP)-conjugated secondary antibody was obtained from GE Healthcare Life Sciences (Pittsburgh, PA, USA).

### Immunoblot analysis

After washing with PBS, cells were lysed in RIPA buffer (Thermo Scientific, Waltham, MA, USA) supplemented with 1% protease/phosphatase inhibitor cocktail (Cell Signaling Technology). Protein concentrations were measured using the bicinchoninic acid method (Thermo Scientific). The cell lysates were separated on 4–12% Bis-Tris gels (Life Technologies) and transferred to polyvinylidene difluoride (PVDF) membranes (Nippon Genetics, Tokyo, Japan). After blocking with 5% skim milk in Tris-buffered saline supplemented with Tween20 (TBS-T) at room temperature, the membranes were incubated with primary antibodies in Can Get Signal solution 1 (Toyobo Life Science, Tokyo, Japan) at 4 °C overnight, followed by incubation with secondary antibodies in Can Get Signal solution 2 (Toyobo Life Science) at room temperature for 1 h. After washing with TBS-T, immunoreactive bands were visualized using chemiluminescent reagents (ECL prime; GE Healthcare Life Sciences and ImmunoStar LD; Wako, Osaka, Japan).

### RNA interference

Lipofectamine 2000 (Life Technologies) was used to transfect cells with 20 nM siRNAs, according to the manufacturer’s instruction. Two kinds of siRNAs targeting c-MET (constructs I and II; #6618 and #6622) and a non-targeting siRNA (#6568) were purchased from Cell Signaling Technology, Inc. Two kinds of siRNAs targeting PDGFRα (Hs_PDGFRα_1109, 6393) and a non-targeting siRNA (SIC-001) were obtained from Sigma-Aldrich.

### Cell proliferation assay

SS cells were plated in 96-well plates for cell proliferation assays. The cell proliferation rate was measured using the premixed WST-1 cell proliferation assay system (Takara Bio, Inc., Otsu, Japan). The relative cell proliferation rate was calculated by subtracting absorbance measurements obtained from a microplate reader at 690 nm from those obtained at 450 nm.

### Cell cycle analysis

SS cells (5 × 10^5^ per dish) were seeded in 10-cm culture dishes and incubated overnight and then treated with TAS-115 or control (DMSO) for 24 h. The cells were harvested and stained with Propidium Iodide (PI) solution (25 μg/ml PI, 0.03% NP-40, 0.02 mg/ml RNase A, 0.1% sodium citrate) for 30 min at room temperature. We analysed the cell cycle using a BD FACSCanto II flow cytometer (Becton Dickinson (BD) Biosciences, San Jose, CA, USA).

### In vivo xenograft experiments

The animal studies were performed in accordance with a guideline approved by the Institutional Animal Care and Use Committee of the Osaka University Graduate School of Medicine. We used Yamato-SS and SYO-1 cells because of their consistency in producing tumours in xenograft models. Yamato-SS cells (3 × 10^7^) or SYO-1 cells (1 × 10^7^) were inoculated subcutaneously into the flank of 5-week-old male BALB/c nu/nu mice (SLC, Shizuoka, Japan). Tumour volume (mm^3^) was defined as (A × B^2^)/2, where A and B were the longest and the shortest diameter of the tumour, respectively. Oral administration of TAS-115 was initiated after the average size of the established tumours reached around 100 mm^3^. TAS-115 was administered once daily at a dose of 50 or 200 mg/kg for 4 weeks. Xenograft tumour volume and the body weight of mice were measured once a week. After 4 weeks of treatment, the mice were euthanized and the tumour weight was measured. Resected tumours were used for immunohistochemical studies.

For immunoblot analyses, mice bearing tumours were orally treated with TAS-115 (200 mg/kg) and with pazopanib (100 mg/kg) for consecutive 3 days. Three hours after the last drug administration, the tumours were resected and extracted in Tissue Protein Extraction Reagent (Thermo Scientific). The dose of TAS-115 or pazopanib was determined based upon prior reports in which daily administration of TAS-115 (50–200 mg/kg) or pazopanib (100 mg/kg) resulted in significant growth inhibition of the xenograft tumours [15, 16, 22, 23].

### Immunohistochemistry

Xenograft tumours were fixed in 10% neutral-buffered formalin and embedded in paraffin. Sections (4 μm) were deparaffinized and dehydrated. After antigen retrieval in 10-mM citrate buffer at 95 °C for 30 min, endogenous peroxidase activity was blocked with methanol containing 3% H_2_O_2_ for 10 min. The sections were incubated with primary antibodies at 4 °C overnight, with secondary antibodies for 1 h on the next day, and then stained with 3,3′-diaminobenzidine tetrahydrochloride (DAB; Dako, Glostrup, Denmark). Finally, these sections were counterstained with hematoxylin.

### Measurement of PCNA positive rate

PCNA positive rate was assessed by counting >500 cells from 3 random fields of each specimen under × 200 magnification in the best-stained tumour area of each section.

### Statistical analysis

We used Student’s *t*-tests for experiments in vitro and the Mann–Whitney U test for experiments in vivo. Values of *p* < 0.05 were considered statistically significant.

## Results

### SS cell lines could be divided into two groups: C-MET-dependent and PDGFRα-dependent SS cells

**Fig. 1:**
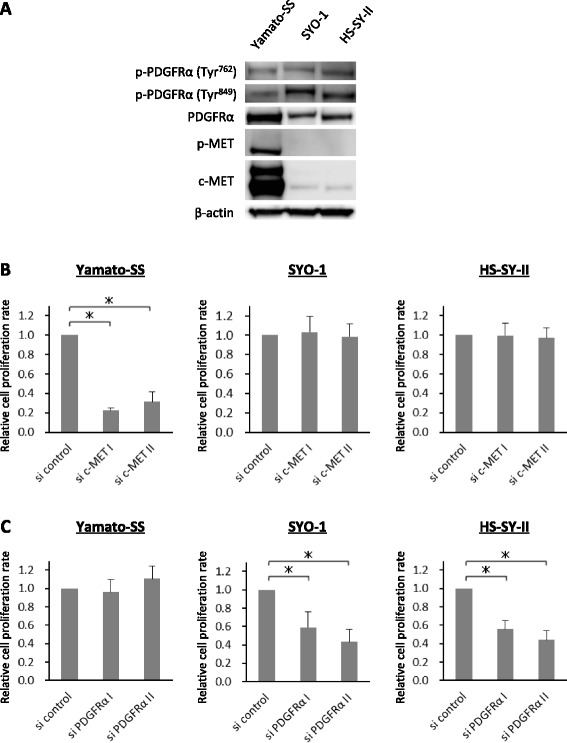
c-MET and PDGFRα signals are crucial for the proliferation of SS cells. **a** Phosphorylation status of RTKs in 3 SS cell lines. **b** Yamato-SS (3 × 10^3^), SYO-1 (5 × 10^3^) and HS-SY-II (1 × 10^4^) cells were transfected with siRNAs against c-MET. Transfected cells were cultured for 96 h and relative cell proliferation rates were measured using a WST-1 assay. *Bars* represent the SD. * *p* < 0.05. **c** Growth of Yamato-SS (3 × 10^3^), SYO-1 (5 × 10^3^) and HS-SY-II (1 × 10^4^) cells transfected with siRNAs against PDGFRα. Relative cell proliferation rates were determined using a WST-1 assay after 96 h. *Bars* represent the SD. * *p* < 0.05

We first performed immunoblot analyses to evaluate the phosphorylation status of RTKs in the Yamato-SS, SYO-1 and HS-SY-II cell lines. Immunoblot analyses revealed that c-MET was activated in Yamato-SS cells, whereas PDGFRα was activated in all three SS cell lines (Fig. 1a).

Next, we used RNA interference technology to determine which RTK was crucial for the viability of the Yamato-SS, SYO-1 and HS-SY-II cell lines. Two kinds of small interfering RNAs (siRNAs) against c-MET or those against PDGFRα were transfected into Yamato-SS, SYO-1 and HS-SY-II cells. Silencing of c-MET expression significantly inhibited the growth of Yamato-SS cells but had little effect on the viability of the SYO-1 or HS-SY-II cells (Fig. 1b and Additional file 1: Figure S1A). By contrast, knockdown of PDGFRα expression markedly abrogated the proliferation of SYO-1 and HS-SY-II cells, but not that of Yamato-SS cells (Fig. 1c and Additional file 1: Figure S1B). These results suggested that Yamato-SS cell proliferation was highly addicted to the c-MET signalling pathway, whereas the proliferation of SYO-1 or HS-SY-II cells was dependent upon the PDGFRα signalling pathway.

### TAS-115 suppresses the growth of both c-MET-dependent and PDGFRα-dependent SS cells in vitro

**Fig. 2:**
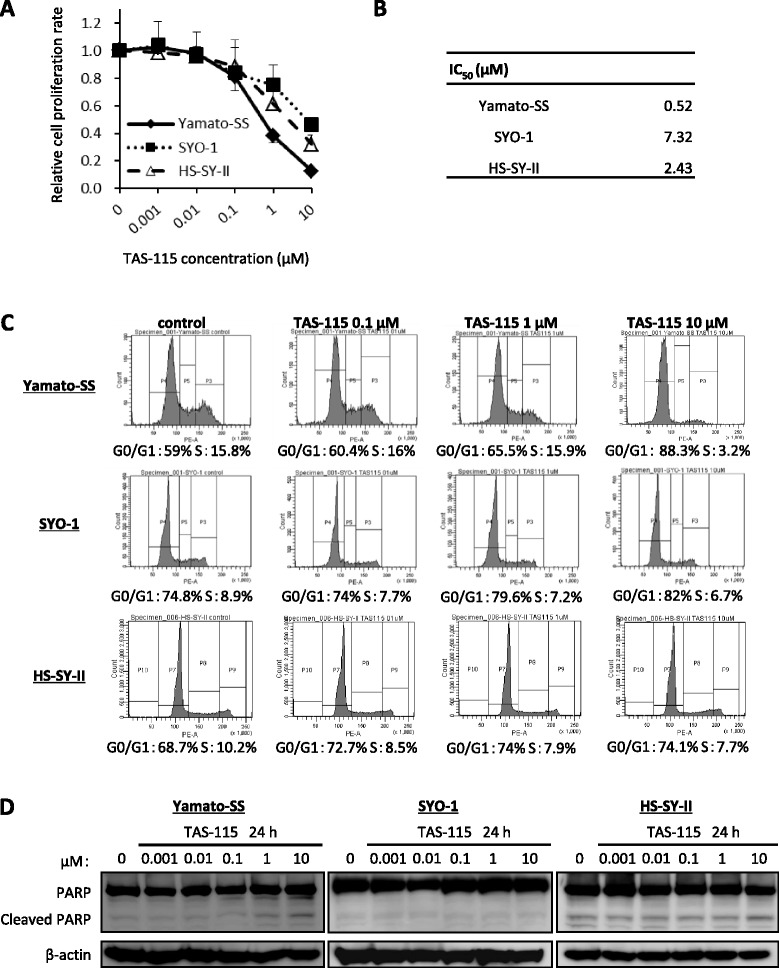
TAS-115 inhibits the growth of SS cells by inducing G0/G1 cell cycle arrest and apoptosis. **a** Yamato-SS, SYO-1 and HS-SY-II cells (3 × 10^3^) were treated with TAS-115 in a concentration range of 0 to 10 μM for 72 h. Relative cell proliferation rates were determined using a WST-1 assay. *Bars* represent the SD. **b** Calculated IC_50_ values of each cell line. **c** The effect of TAS-115 on the cell cycle. Yamato-SS, SYO-1 and HS-SY-II cells were treated with control (0.1% DMSO) or 0.1–10-μM TAS-115 for 24 h. After treatment, the cells were stained with PI solution for flow cytometric analysis. **d** The effect of TAS-115 on PARP cleavage in Yamato-SS, SYO-1 and HS-SY-II cells. Cells were treated control (0.1% DMSO) or 0.001–10 μM of TAS-115 for 24 h

We performed WST-1 cell proliferation assays to examine the antitumour activity of TAS-115, known as a c-MET/VEGFR dual tyrosine kinase inhibitor, against SS cell lines in vitro. TAS-115 inhibited the growth of all three SS cell lines in a dose-dependent manner (Fig. 2a). In addition, the 50% inhibitory concentrations (IC_50_s) of TAS-115 in the Yamato-SS, SYO-1 and HS-SY-II cell lines were 0.52, 7.32 and 2.43 μM, respectively (Fig. 2b). These results suggested that the Yamato-SS cells, which were c-MET-dependent SS cells, were more sensitive to TAS-115 than the SYO-1 and HS-SY-II cells, which were PDGFRα-dependent SS cells.

Flow cytometric analyses were conducted to elucidate the mechanisms by which TAS-115 inhibited SS cell proliferation. TAS-115 increased the percentage of cells in the G0/G1-phase and decreased the percentage of cells in the S-phase in the Yamato-SS, SYO-1 and HS-SY-II cells in a dose-dependent manner (Fig. 2c). Immunoblot analyses revealed that levels of cleaved poly ADP ribose polymerase (PARP) mildly increased in the Yamato-SS cells, whereas PARP cleavage did not occur in either the SYO-1 or HS-SY-II cells after treatment with TAS-115 for 24 h (Fig. 2d). These observations indicated that TAS-115 suppressed cell proliferation by inducing G0/G1 cell cycle arrest in all SS cell lines. TAS-115 also caused slight apoptosis in the Yamato-SS cells, but not in the SYO-1 or HS-SY-II cells.

### TAS-115 blocks phosphorylation of PDGFRα and c-MET, as well as their downstream effectors in vitro

**Fig. 3:**
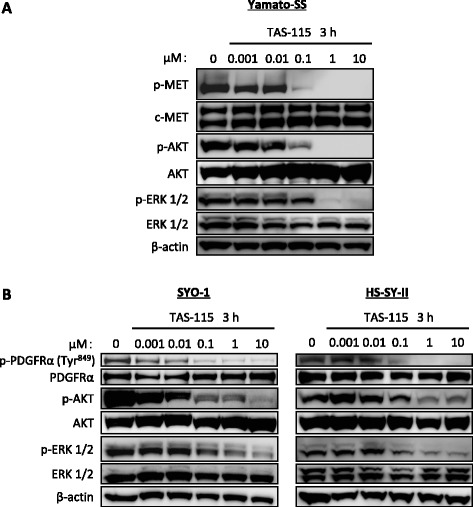
TAS-115 suppresses phosphorylation of c-MET and PDGFRα, as well as their downstream effectors. **a** Yamato-SS cells (c-MET-dependent SS cells) were treated with 0.001–10 μM of TAS-115 or control (0.1% DMSO) for 3 h. **b** SYO-1 and HS-SY-II cells (PDGFRα-dependent SS cells) were treated with 0.001–10 μM of TAS-115 or control (0.1% DMSO) for 3 h

We investigated the effects of TAS-115 on the c-MET and PDGFRα signalling pathways by immunoblot analyses in vitro. c-MET phosphorylation was markedly suppressed in Yamato-SS cells after a 3-h incubation with TAS-115 at concentrations as low as 0.1 to 10 μM. Moreover, treatment with TAS-115 at these concentrations also inhibited the phosphorylation of downstream effectors, such as AKT and extracellular signal-regulated kinase (ERK) 1/2 (Fig. 3a).

PDGFRα has been reported to have more than 10 autophosphorylation sites. Among them, the PDGFRα residue Tyr^849^, which is located inside tyrosine kinase domain II, is critical for activation of the kinase [24]. Thus, we first focused on this tyrosine residue. TAS-115 at concentrations as low as 1–10 μM remarkably inhibited the phosphorylation of PDGFRα on Tyr^849^, as well as its downstream effectors AKT and ERK 1/2, in the SYO-1 and HS-SY-II cells (Fig. 3b). In rhPDGF-BB-stimulated SYO-1 and HS-SY-II cells, TAS-115 at concentrations of 0.01 μM or higher remarkably suppressed the phosphorylation of PDGFRα at Tyr^849^, as well as its downstream effectors (Additional file 2: Figure S2). These results indicated that TAS-115 inhibited the activity of PDGFRα and strongly suppressed c-MET phosphorylation.

### Comparing the inhibitory effects of TAS-115 and pazopanib on c-MET or PDGFRα phosphorylation

**Fig. 4:**
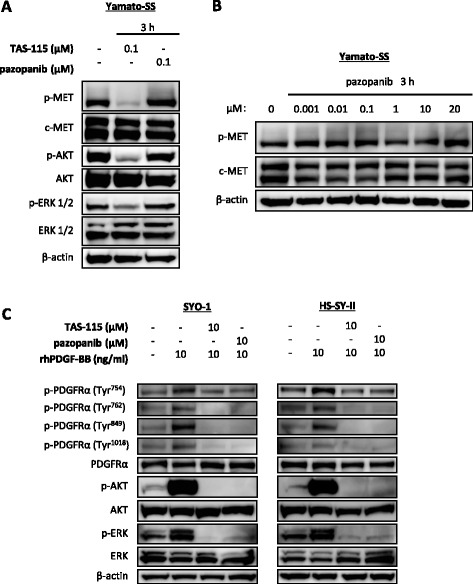
Inhibitory activities of TAS-115 and pazopanib on c-MET, PDGFRα and their downstream effectors in vitro. **a** Yamato-SS cells (c-MET-dependent SS cells) were treated with 0.1 μM TAS-115 or pazopanib or control (0.1% DMSO) for 3 h. **b** Yamato-SS cells were treated with 0.001–20 μM of pazopanib or control (0.1% DMSO) for 3 h. **c** SYO-1 and HS-SY-II (PDGFRα-dependent) SS cells were treated with 10-μM TAS-115 or pazopanib or control (0.1% DMSO) for 3 h, followed by an additional treatment with rhPDGF-BB at a concentration of 10 ng/ml for the last 15 min

We compared the abilities of TAS-115 and pazopanib to inhibit the c-MET and PDGFRα pathways using immunoblot analyses. TAS-115 at a concentration of 0.1 μM suppressed c-MET, AKT and ERK 1/2 phosphorylation, whereas pazopanib at the same concentration inhibited neither c-MET phosphorylation nor the phosphorylation of downstream effectors in the Yamato-SS cells (Fig. 4a). When increasing the drug concentration from 0.001 to 20 μM, pazopanib had no demonstrable effect on the phosphorylation of c-MET in the Yamato-SS cells (Fig. 4b).

In SYO-1 and HS-SY-II cells, rhPDGF-BB treatment enhanced the phosphorylation of PDGFRα at Tyr^754^, Tyr^762^, Tyr^849^, Tyr^1018^, as well as the phosphorylation of downstream effectors, AKT and ERK 1/2. Treatment with 10-μM TAS-115 inhibited phosphorylation at these sites of PDGFRα, which resulted in the subsequent suppression of AKT and ERK 1/2 activity. Pazopanib (10 μM) also attenuated the phosphorylation of these tyrosine residues of PDGFRα and the activity of downstream effectors (Fig. 4c).

**Fig. 5:**
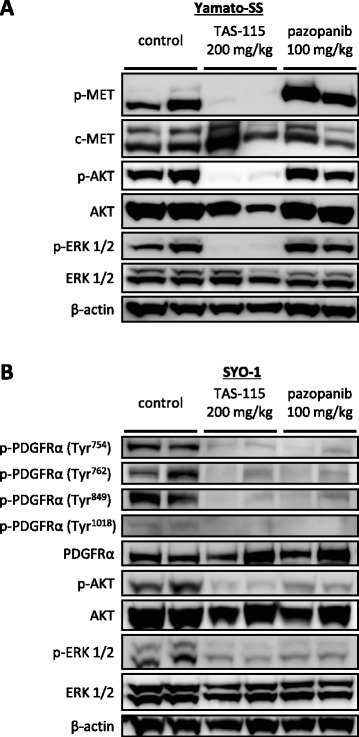
Inhibitory effect of TAS-115 and pazopanib on c-MET, PDGFRα and their downstream effectors in vivo. **a**, **b** Mice bearing Yamato-SS (c-MET-dependent) cells and mice bearing SYO-1 (PDGFRα-dependent) SS cells were treated with orally administered TAS-115 (200 mg/kg) or pazopanib (100 mg/kg) or control for consecutive 3 days and euthanized 3 h after the final administration

To verify the inhibitory effects of TAS-115 and pazopanib on c-MET and PDGFRα in vivo, we administered TAS-115 (200 mg/kg) and pazopanib (100 mg/kg) orally to mice bearing Yamato-SS or SYO-1 xenograft tumours. TAS-115 at a dose of 200 mg/kg inhibited the phosphorylation of c-MET, AKT and ERK 1/2, while pazopanib did not affect c-MET signalling in Yamato-SS xenograft tumours (Fig. 5a). Both TAS-115 and pazopanib blocked the phosphorylation of PDGFRα at Tyr^754^, Tyr^762^, Tyr^849^, Tyr^1018^, as well as the phosphorylation of downstream effectors in SYO-1 xenograft tumours (Fig. 5b). These observations suggested that pazopanib attenuated the phosphorylation of PDGFRα, but not of c-MET. On the other hand, TAS-115 inactivated both c-MET and PDGFRα; moreover, the inhibitory activity of TAS-115 against PDGFRα phosphorylation was probably equivalent to that of pazopanib with respect to at least four representative autophosphorylation sites of the receptor in vitro and in vivo.

### TAS-115 abrogates the growth of Yamato-SS and SYO-1 xenograft tumours

**Fig. 6:**
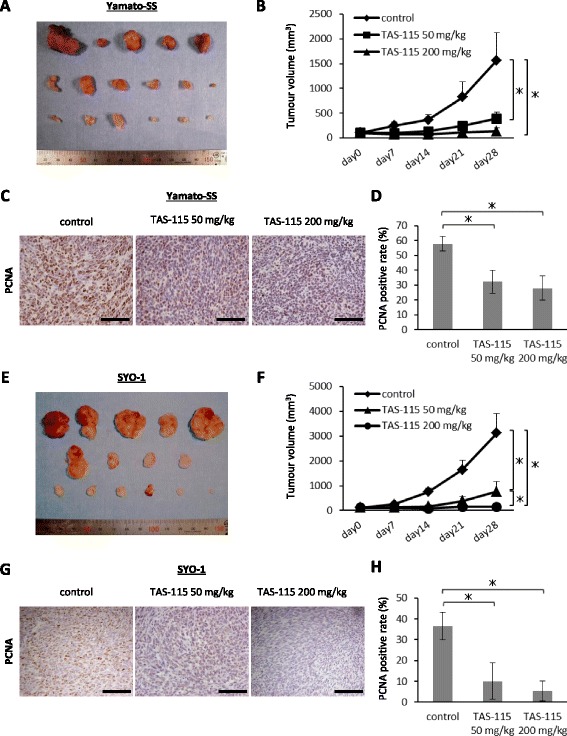
TAS-115 strongly abrogates the growth of Yamato-SS and SYO-1 xenograft tumours. **a** The appearance of resected Yamato-SS tumours at the end of the experiments. **b** Mice bearing Yamato-SS xenografts were treated with 50 or 200 mg/kg of TAS-115, or control. *Bars* represent the SE. * *p* < 0.05. **c** Immunohistological analysis of PCNA (× 200). Scale bars, 100 μm. **d** PCNA-positive rate of each treatment group. *Bars* represent the SD. * *p* < 0.05. **e** The appearance of resected SYO-1 tumours at the end of the experiments. **f** Mice bearing SYO-1 xenografts were treated with 50 or 200 mg/kg of TAS-115, or control. *Bars* represent the SE. * *p* < 0.05. **g** Immunohistological analysis of PCNA (× 200). Scale bars, 100 μm. **h** PCNA-positive rate of each treatment group. *Bars* represent the SD. * *p* < 0.05

We tested the antitumour effect of TAS-115 against Yamato-SS (c-MET-dependent SS cells) and SYO-1 (PDGFRα-dependent SS cells) xenograft tumours. Mice bearing tumours were treated daily with an oral dose of TAS-115 at 50 or 200 mg/kg, or a control. TAS-115 at a dose of 50 mg/kg showed moderate inhibitory activity for Yamato-SS xenograft tumours. Notably, treatment with 200 mg/kg of TAS-115 completely prevented the tumour growth during the treatment period (Fig. 6a, b). A remarkable decrease in tumour wet weight was observed in tumours treated with TAS-115 at a dose of 200 mg/kg (Additional file 3: Figure S3). No marked body weight loss was observed in TAS-115-treated mice (Additional file 4: Figure S4). Immunoblot analyses of resected tumours demonstrated that TAS-115 treatment inhibited the phosphorylation of c-MET, AKT and ERK 1/2 (Additional file 5: Figure S5). Histological analyses showed a decrease in the density of the tumour cells, as well as slight myxoid degeneration, with no signs of an inflammatory reaction or necrosis in TAS-115 treated groups (Additional file 6: Figure S6A). Additionally, Yamato-SS xenograft tumours sustained both spindle and epithelial cells after 28-day treatment in all treatment groups (Additional file 6: Figure S6A), indicating that TAS-115 might have similar effect on both spindle and epithelial components. Immunohistochemical analyses revealed that the number of proliferating cell nuclear antigen (PCNA)-positive tumour cells was significantly reduced in Yamato-SS xenograft tumours treated with TAS-115 (Fig. 6c, d). Besides, TAS-115 dose-dependently inhibited microvascular density (MVD) (Additional file 7: Figure S7A, B and Additional file 8: Supplementary methods). Unlike our in vitro experiments, cleaved caspase-3 was not detected in the Yamato-SS xenografts (data not shown). Apoptosis did not seem to be involved in the antitumour mechanisms of TAS-115 in vivo.

Similarly to experiments in the Yamato-SS xenografts, TAS-115 administration showed mild inhibition (50 mg/kg) and entire suppression (200 mg/kg) of the tumour growth and wet weight in SYO-1-transplanted mice (Fig. 6e, f and Additional file 3: Figure S3). Body weight loss was not observed in these TAS-115-treated mice (Additional file 4: Figure S4), either. Tumours treated with TAS-115 also showed a decrease in the cell density along with slight myxoid changes (Additional file 6: Figure S6B). As seen in Yamato-SS xenograft models, not only spindle cells but also epithelial tumour cells were observed in SYO-1 xenograft tumours after TAS-115 administration (Additional file 6: Figure S6B), which was confirmed by immunohistochemical staining of anti-vimentin and anti-cytokeratin (AE1/AE3) antibodies (Additional file 9: Figure S8 and Additional file 8: Supplementary methods). Treatment with TAS-115 also resulted in a significant reduction in PCNA-positive tumour cells and MVD in SYO-1 xenografts (Fig. 6g, h and Additional file 7: Figure S7C, D and Additional file 8: Supplementary methods).

These results suggested that TAS-115 exhibited strong antitumour effects for both c-MET-dependent and PDGFRα-dependent SS cells in vivo by inhibiting the proliferation of tumour cells, as well as tumour vascular development, without any demonstrable adverse events.

## Discussion

Aberrant activation of HGF/c-MET signalling by mutation, autocrine or paracrine HGF stimulation, or overexpression has been implicated in the oncogenesis of large number of cancers [10, 25–28]. Additionally, upregulation or mutational activation of PDGF ligand or PDGFR expression has also been reported in many types of malignancies [12, 29–31]. Co-expression of HGF and c-MET has been seen in 14% of SS clinical samples, correlating with poor prognosis [32]. Likewise, we noted that 36% of SS specimens expressed both HGF and c-MET, which resulted in a significantly worse clinical course in SS patients [11]. A recent study revealed that PDGF-AA expression and phosphorylation of PDGFRα without any gene alteration led to AKT activation through an autocrine/paracrine-mediated loop in a subset of clinical SS tumour samples [33]. In the present study, we found c-MET activation in the Yamato-SS cells and PDGFRα phosphorylation in all three SS cell lines. Silencing of c-MET or PDGFRα expression revealed that the proliferation of the Yamato-SS cells sustained the high dependency upon c-MET signalling and that the viability of the SYO-1 or HS-SY-II cells was primarily driven by PDGFRα pathway. Based upon these observations, we could divide the SS cell lines tested in this study into two groups: c-MET-dependent SS cells (Yamato-SS) and PDGFRα-dependent SS cells (SYO-1 and HS-SY-II).

RTK cascades appear to be the principal activators of intracellular signalling through the PI3K/AKT and mitogen-activated protein kinase (MAPK) pathways. These downstream pathways have been reported as critical to SS viability [34, 35]. TAS-115, a novel c-MET/VEGFR-targeting dual tyrosine kinase inhibitor, has been reported to inhibit multiple RTKs besides c-MET and VEGFR under cell-free conditions [15]. TAS-115 significantly suppressed the proliferation of both c-MET-dependent and PDGFRα-dependent SS cells, mainly by inducing G0/G1 cell cycle arrest. Treatment with TAS-115 attenuated c-MET signalling in Yamato-SS cells, leading to subsequent suppression of AKT and ERK 1/2 phosphorylation. Similarly, blocking PDGFRα function with TAS-115 decreased intracellular signalling in SYO-1 and HS-SY-II cells. Interestingly, the TAS-115 concentration at which c-MET or PDGFRα signalling was inhibited in each cell line, using immunoblot analyses, corresponded to the IC_50_ value for TAS-115 against c-MET-dependent cells (Yamato-SS) or PDGFRα-dependent cells (SYO-1 or HS-SY-II) in WST-1 cell proliferation assays. In agreement with our in vitro findings, TAS-115 exerted anti-c-MET or anti-PDGFRα pathway activity and inhibited their downstream effectors in the Yamato-SS (c-MET-dependent) and SYO-1 (PDGFRα-dependent) cells in in vivo xenograft models. These insights indicate that the c-MET and PDGFRα cascades are the primary regulators of both the PI3K/AKT and MAPK pathways in c-MET-dependent and PDGFRα-dependent SS cells, respectively. This difference of signal dependency in SS cell lines may be ascribed to the diversity among individual tumours. Another possible explanation is an artificial selection of cells with activated c-MET or PDGFRα from heterogeneous cell populations within a tumour during cell-line establishment.

Pazopanib, a PDGFRα and PDGFRβ/ VEGFR/ c-kit-targeting tyrosine kinase inhibitor [13], has been approved by Food and Drug Administration (FDA) for soft tissue sarcoma and renal cell carcinoma [36, 37]. In Japan, this drug is the only tyrosine kinase inhibitor available for treatment of advanced soft tissue sarcomas. Accumulating data suggested that high plasma concentrations of HGF were significantly correlated with shorter progression-free survival in patients treated with pazopanib for metastatic renal cell carcinoma [38, 39]. These clinical studies give rise to an idea that the effect of pazopanib on cancers with active HGF/c-MET signalling might be limited. In the SS cell lines tested, pazopanib attenuated only PDGFRα signalling, while c-MET phosphorylation was not inhibited even at high concentration of pazopanib. By contrast, TAS-115 successfully suppressed both the c-MET and PDGFRα pathways. Moreover, TAS-115 treatment inhibited the phosphorylation of PDGFRα on at least four representative autophosphorylation sites of the receptor, as well as its downstream effectors, equivalently to pazopanib. These data suggest that TAS-115 will have therapeutic capability in SS with active c-MET or PDGFRα pathways, via inhibition of c-MET and PDGFRα signalling, indicating that it might be clinically applicable.

Recently, several investigators argued the possibility of personalized therapy by evaluating the specific signalling activation state of tyrosine kinases in different types of malignancies, including lung carcinoma [40], colorectal carcinoma [41] and ovarian carcinoma [42]. Most recently, we have reported that c-MET phosphorylation in clinical samples is a potential biomarker predicting response to a selective c-MET inhibitor for SS patients [11]. Treatment with a specific tyrosine kinase inhibitor selected by predicting therapeutic efficiency may prevent or delay non-specific deleterious side effects [43]. On the other hand, the possible advantages of a multi-targeting tyrosine kinase inhibitor include simplicity and capability of its use in daily practice for tumours with multiple signalling pathways. The tumours found in patients may have inter-individual differences in the activation states of RTKs that are involved in tumour growth or progression, even if they belong to a pathologically identical group. Moreover, the individual tumour may be a heterogeneous mixture of cell populations driven by multiple RTK signals. Indeed, our results indicated that monotherapy with TAS-115 suppressed both c-MET and PDGFRα signalling, resulting in strong therapeutic effects against two types of SS cells both in vitro and in vivo without outstanding side effects*.* These observations support the view that inhibition of both c-MET and PDGFRα with a single agent is a practical consideration for SS treatment.

The cellular origins of SS are still unknown. While several potential cellular origins of SS have been reported, such as cells of a myogenic or neurogenic lineage [44–46], we have claimed that a multipotent mesenchymal stem cell may be a cell of origin for SS [19]. In this study, we noted the activation of c-MET or PDGFRα and the importance of these signals for viability in SS cell lines. In some translocation-related sarcomas, fusion genes are assumed to contribute to the upregulation of RTK cascades. For instance, c-MET was identified as a direct target of *PAX3-FOXO1* in alveolar rhabdomyosarcoma and *EWS-ATF1* in clear cell sarcoma [27, 47]. Additionally, *EWS-FLI1* directly upregulated *PDGF-C* expression, which in turn activated the PDGFRα pathway in Ewing sarcoma [48]. Correspondingly, *EWS-WT1*, which underlay desmoplastic small round cell tumours, induced *PDGF-A* expression [49]. By sharp contrast, our parallel study showed that *SS18-SSX* silencing did not elicit the downregulation or inactivation of either c-MET or PDGFRα [11]. These data suggest that the activation of c-MET or PDGFRα might not be evoked by *SS18-SSX.* Rather, it reflects a prerequisite cellular background to be permissive for *SS18-SSX*. It has been reported that the window of permissive cells might be relatively narrow for *SS18-SSX* [44, 50]. Thus we speculated that SS might arise from a multipotent mesenchymal stem cell natively driven by HGF/c-MET or PDGF/PDGFR signalling [8]. These findings raise the possibility that SS inherits phosphorylated RTKs from a multipotent mesenchymal stem cell at a certain phase of differentiation and that these RTKs are suitable therapeutic targets for SS. Taken together, RTKs innately present in cells of origin, in addition to those epigenetically altered by fusion genes, might be plausible target molecules for the treatment of translocation-related sarcomas.

Although the findings detailed above do seem clear, there are some limitations in this study. First, since an environment in which either cultured cells or mouse xenografts grow is significantly different from that of their originating tumour, those model systems cannot always provide useful information to generate clinically relevant treatments. Second, in the clinical setting, distant metastases have been reported to be strongly associated with worse outcome of patients with SS [3, 5, 7]. However, experimental models of human SS with spontaneous metastatic potential have not been successfully developed. Thereby, whether TAS-115 monotherapy is sufficient to treat metastatic SS remains unknown. In October 2016, olaratumab, a monoclonal antibody of PDGFRα, received FDA approval for clinical use in combination with doxorubicin for unresectable and metastatic soft tissue sarcomas based upon the clinical data that olaratumab plus doxorubicin showed gains in median progression free and overall survival as compared to doxorubicin alone [51]. Those results lead us to hypothesize that combined therapy of conventional chemotherapeutic agents and TAS-115 targeting PDGFRα and c-MET may be effective for metastatic SS and warrants further investigations. Third, though treatment of TAS-115, introduced as an angiogenesis inhibitor via VEGFR signalling [15], decreased MVD in our in vivo experiments, the extent to which VEGFR inhibition by TAS-115 in the tumour microenvironment contributed to suppression of tumour growth was unclear in this study. Since HGF/c-MET as well as PDGF/PDGFR systems have been recognised as important mediators of angiogenesis [10, 12, 52], it could not be excluded that the antiangiogenic mechanisms of TAS-115 in SS at least in partly encompassed targeting c-MET or PDGFRα. Further clarification would be required in order to make definitive answers to these challenging issues in the future.

## Conclusions

In conclusion, c-MET and PDGFRα signalling are essential for the growth and/or survival of SS tumours. By inhibiting c-MET or PDGFRα signalling, TAS-115 achieves significant therapeutic effects in both c-MET-dependent and PDGFRα-dependent SS cells in vitro and in vivo. Our observations provide strong evidence that TAS-115 can serve as a multiple tyrosine kinase inhibitor which can impede c-MET and PDGFRα signalling and may offer the distinct clinical advantages even when used as a monotherapy for patients with SS tumours driven by either c-MET or PDGFRα pathways.

## Additional files

Additional file 1: Figure S1. (A) Expression of c-MET in Yamato-SS, SYO-1 and HS-SY-II cells after treatment with anti-c-MET siRNAs or a control siRNA. (B) PDGFRα expression in Yamato-SS, SYO-1 and HS-SY-II cells after treatment with anti-PDGFRα siRNAs or a control siRNA. (PPTX 166 kb)

Additional file 2: Figure S2. SYO-1 and HS-SY-II (PDGFRα-dependent) SS cells were treated with 0.001–10 μM of TAS-115 or control (0.1% DMSO) for 3 h, followed by an additional treatment with 10-ng/ml rhPDGF-BB for the last 15 min. (PPTX 178 kb)

Additional file 3: Figure S3. The weight of Yamato-SS and SYO-1 xenograft tumours for each treatment group. Bars represent the SE. * *p* < 0.05. N.S., not significant. (PPTX 58 kb)

Additional file 4: Figure S4. Body weight of mice bearing Yamato-SS and SYO-1 cells for each treatment group. Bars represent the SE. (PPTX 64 kb)

Additional file 5: Figure S5. Immunoblot analysis of Yamato-SS xenografts. Mice bearing Yamato-SS cells were treated with 50 or 200-mg/kg TAS-115 or control orally once a day for 4 weeks, euthanized 3 h after final administration and subjected to immunoblot analysis. (PPTX 117 kb)

Additional file 6: Figure S6. (A) Light microscopic findings of Yamato-SS xenograft tumours for each treatment group (× 200). Scale bars, 100 μm. The tumours had both spindle cell components (upper panels) and epithelial cell components (lower panels). (B) Light microscopic findings of SYO-1 xenograft tumours for each treatment group (× 200). Scale bars, 100 μm. The tumours had both spindle cell components (upper panels) and epithelial cell components (lower panels). (PPTX 7168 kb)

Additional file 7: Figure S7. (A) Immunohistological staining of anti-CD31 antibody in Yamato-SS xenograft tumours for each treatment group (× 200). Scale bars, 100 μm. (B) Microvascular density (MVD) of Yamato-SS xenograft tumours. Bars represent the SD. * *p* < 0.05. (C) Immunohistological staining of anti-CD31 antibody in SYO-1 xenograft tumours for each treatment group (× 200). Scale bars, 100 μm. (D) Microvascular density (MVD) of SYO-1 xenograft tumours. Bars represent the SD. * *p* < 0.05. (PPTX 2778 kb)

Additional file 8: Supplementary methods. (DOCX 16 kb)

Additional file 9: Figure S8. Immunohistological staining of anti-vimentin and anti-cytokeratin (AE1/AE3) antibodies in SYO-1 xenograft tumours for each treatment group (× 400). Vimentin was diffusely present in both spindle and epithelial components, whereas epithelial cells expressed cytokeratin (AE1/AE3) relatively higher than spindle tumour cells. Scale bars, 100 μm. (PPTX 7779 kb)

## Abbreviations

c-kit: Stem cell factor receptor
DMSO: Dimethyl sulfoxide
ERK: Extracellular signal-regulated kinase
FDA: Food and Drug Administration
HGF: Hepatocyte growth factor
IC_50_: 50% inhibitory concentration
MAPK: Mitogen-activated protein kinase
MVD: Microvascular density
PARP: Poly ADP-ribose polymerase
PCNA: Proliferating cell nuclear antigen
PDGF: Platelet-derived growth factor
PDGFR: Platelet-derived growth factor receptor
PI3K: Phosphatidylinositol 3-kinase
RTK: Receptor tyrosine kinase
siRNA: small interference RNA
SS: Synovial sarcoma
VEGFR: Vascular endothelial growth factor receptor
