# Supplementary material for: Therapeutic potential of TAS-115 via c-MET and PDGFRα signal inhibition for synovial sarcoma
Source: BMC Cancer. 2017 May 16;17:334. doi: 10.1186/s12885-017-3324-3 (PMC5434537; doi:10.1186/s12885-017-3324-3)
Supplement: Supplementary file 1 — (A) Expression of c-MET in Yamato-SS, SYO-1 and HS-SY-II cells after treatment with anti-c-MET siRNAs or a control siRNA. (B) PDGFRα expression in Yamato-SS, SYO-1 and HS-SY-II cells after treatment with anti-PDGFRα siRNAs or a control siRNA. (PPTX 166 kb) [file 12885_2017_3324_MOESM1_ESM.pptx]

## Slide 1
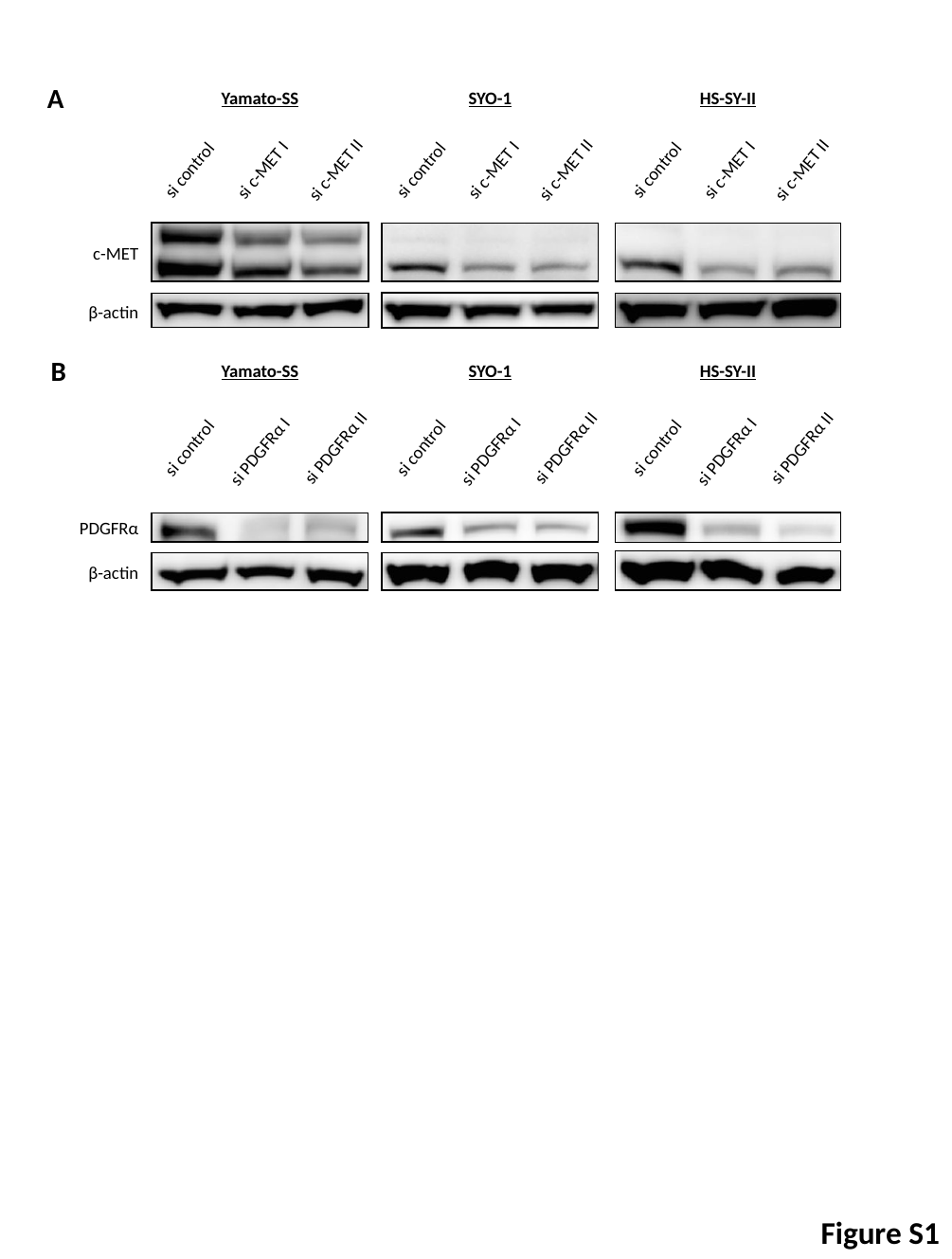

A
Yamato-SS
SYO-1
HS-SY-II
si control
si c-MET I
si c-MET II
si control
si c-MET I
si c-MET II
si control
si c-MET I
si c-MET II
c-MET
β-actin
B
Yamato-SS
SYO-1
HS-SY-II
si control
si PDGFRα I
si PDGFRα II
si control
si PDGFRα I
si PDGFRα II
si control
si PDGFRα I
si PDGFRα II
PDGFRα
β-actin
Figure S1
