# Supplementary figures and images for: Therapeutic potential of TAS-115 via c-MET and PDGFRα signal inhibition for synovial sarcoma
Source: BMC Cancer. 2017 May 16;17:334. doi: 10.1186/s12885-017-3324-3 (PMC5434537; doi:10.1186/s12885-017-3324-3)

## Slide 1
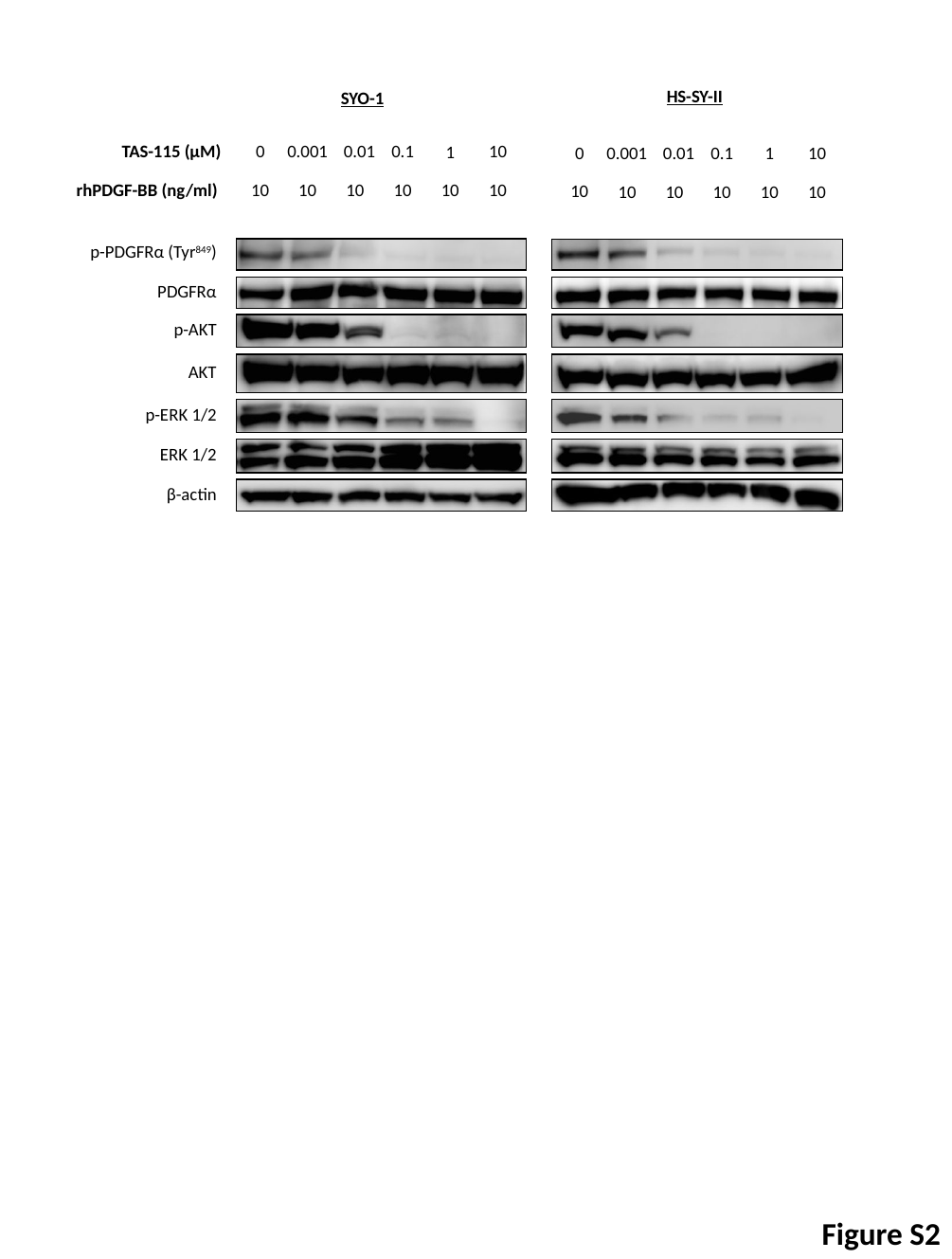

HS-SY-II
SYO-1
 TAS-115 (μM)
0
0.001
0.01
0.1
10
1
0
0.001
0.01
0.1
10
1
rhPDGF-BB (ng/ml)
10
10
10
10
10
10
10
10
10
10
10
10
p-PDGFRα (Tyr849)
PDGFRα
p-AKT
AKT
p-ERK 1/2
ERK 1/2
β-actin
Figure S2

Supplement: Supplementary file 2 — SYO-1 and HS-SY-II (PDGFRα-dependent) SS cells were treated with 0.001–10 μM of TAS-115 or control (0.1% DMSO) for 3 h, followed by an additional treatment with 10-ng/ml rhPDGF-BB for the last 15 min. (PPTX 178 kb) [file 12885_2017_3324_MOESM2_ESM.pptx]

## Slide 1
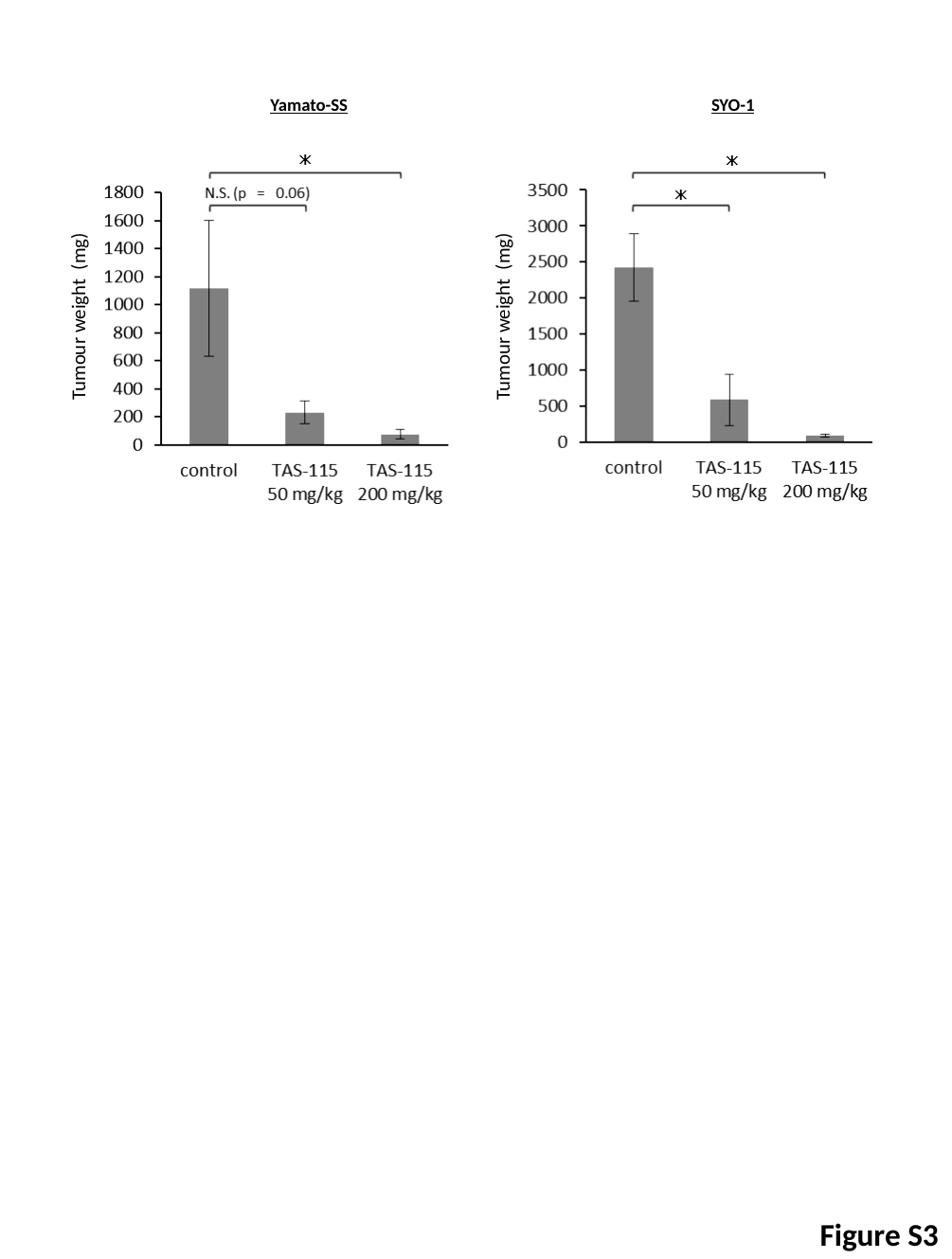

Yamato-SS
SYO-1
Tumour weight (mg)
Tumour weight (mg)
Figure S3

Supplement: Supplementary file 3 — The weight of Yamato-SS and SYO-1 xenograft tumours for each treatment group. Bars represent the SE. * p < 0.05. N.S., not significant. (PPTX 58 kb) [file 12885_2017_3324_MOESM3_ESM.pptx]

## Slide 1
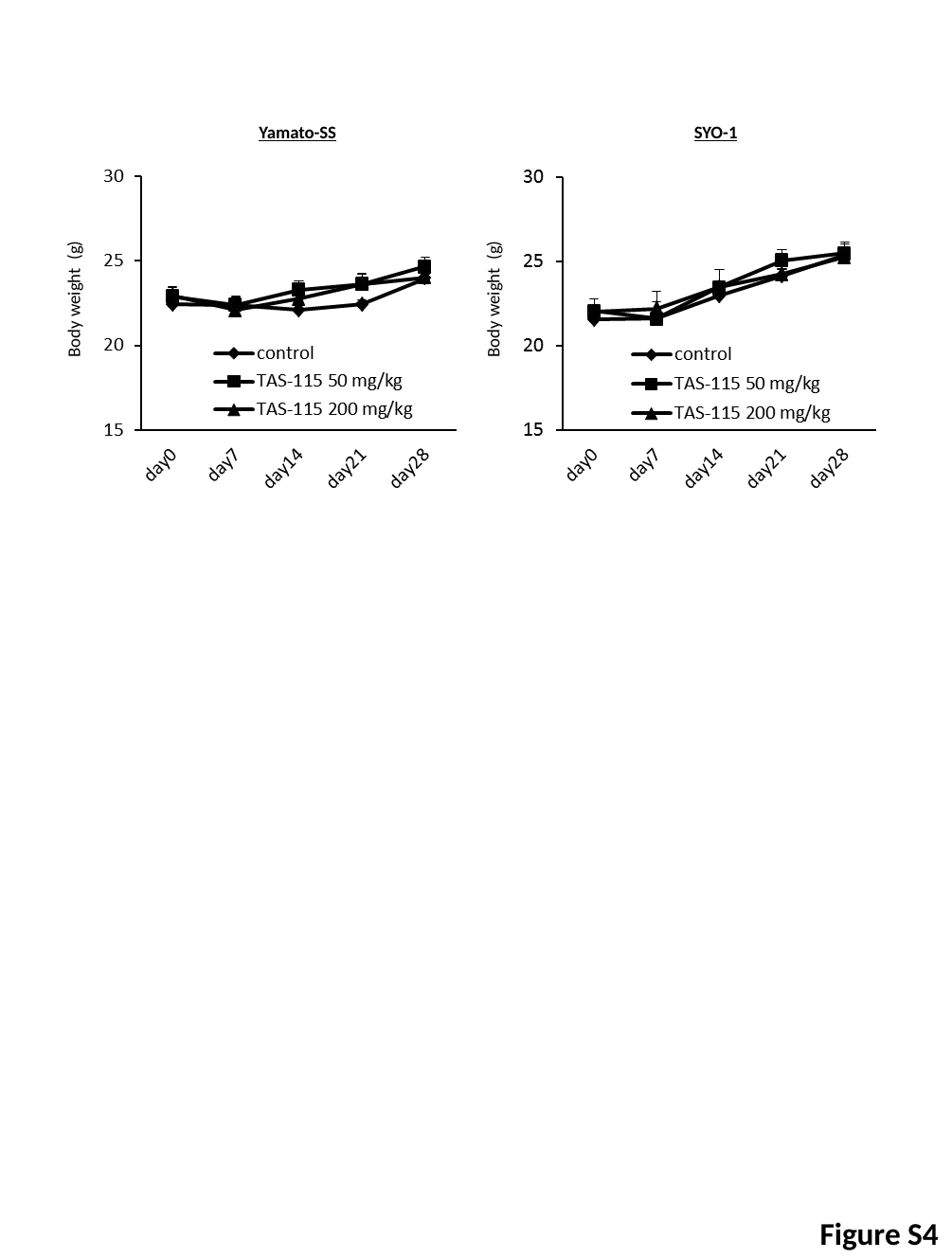

Yamato-SS
SYO-1
Body weight (g)
Body weight (g)
Figure S4

Supplement: Supplementary file 4 — Body weight of mice bearing Yamato-SS and SYO-1 cells for each treatment group. Bars represent the SE. (PPTX 64 kb) [file 12885_2017_3324_MOESM4_ESM.pptx]

## Slide 1
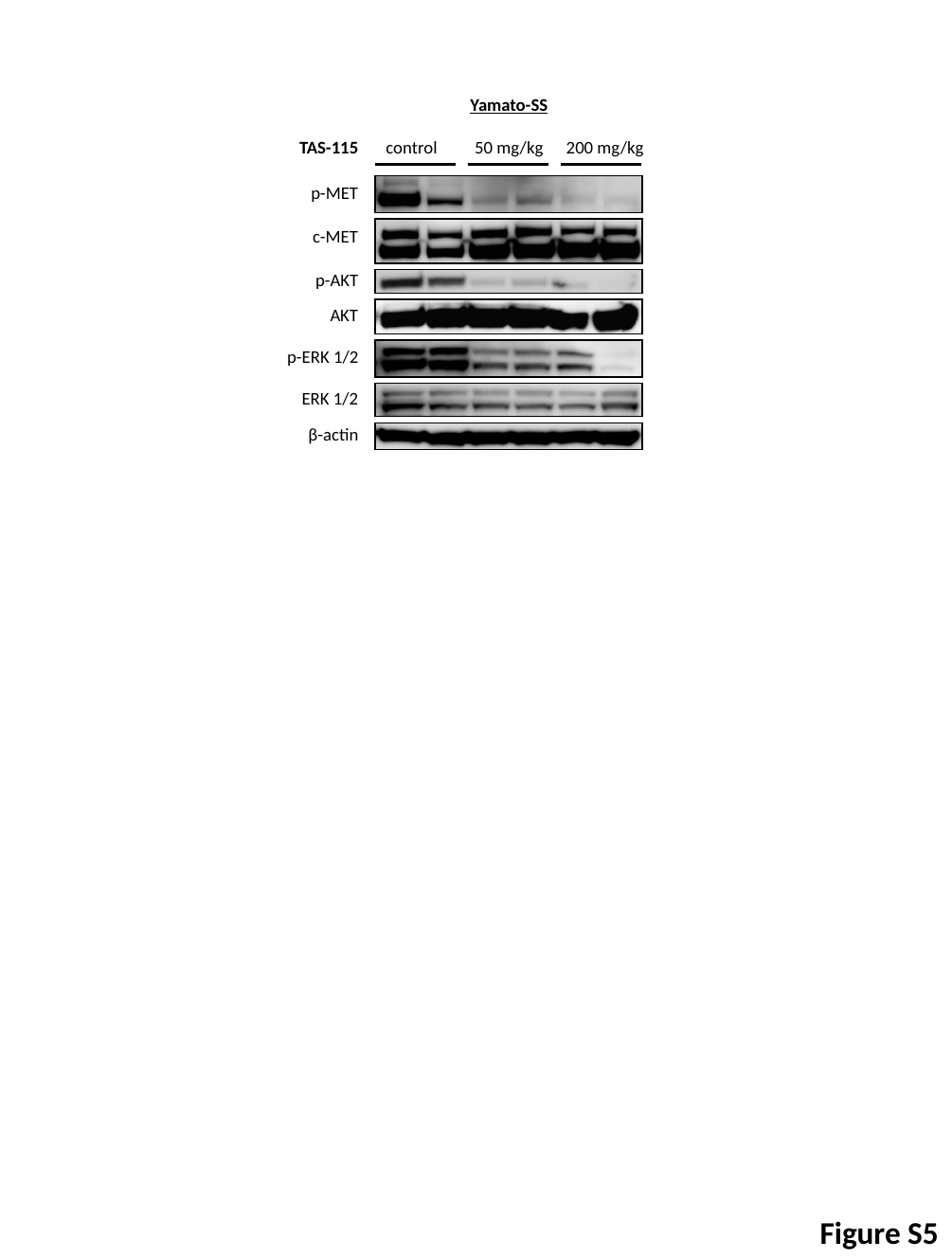

Yamato-SS
TAS-115
control
50 mg/kg
200 mg/kg
p-MET
c-MET
p-AKT
AKT
p-ERK 1/2
ERK 1/2
β-actin
Figure S5

Supplement: Supplementary file 5 — Immunoblot analysis of Yamato-SS xenografts. Mice bearing Yamato-SS cells were treated with 50 or 200-mg/kg TAS-115 or control orally once a day for 4 weeks, euthanized 3 h after final administration and subjected to immunoblot analysis. (PPTX 117 kb) [file 12885_2017_3324_MOESM5_ESM.pptx]

## Slide 1
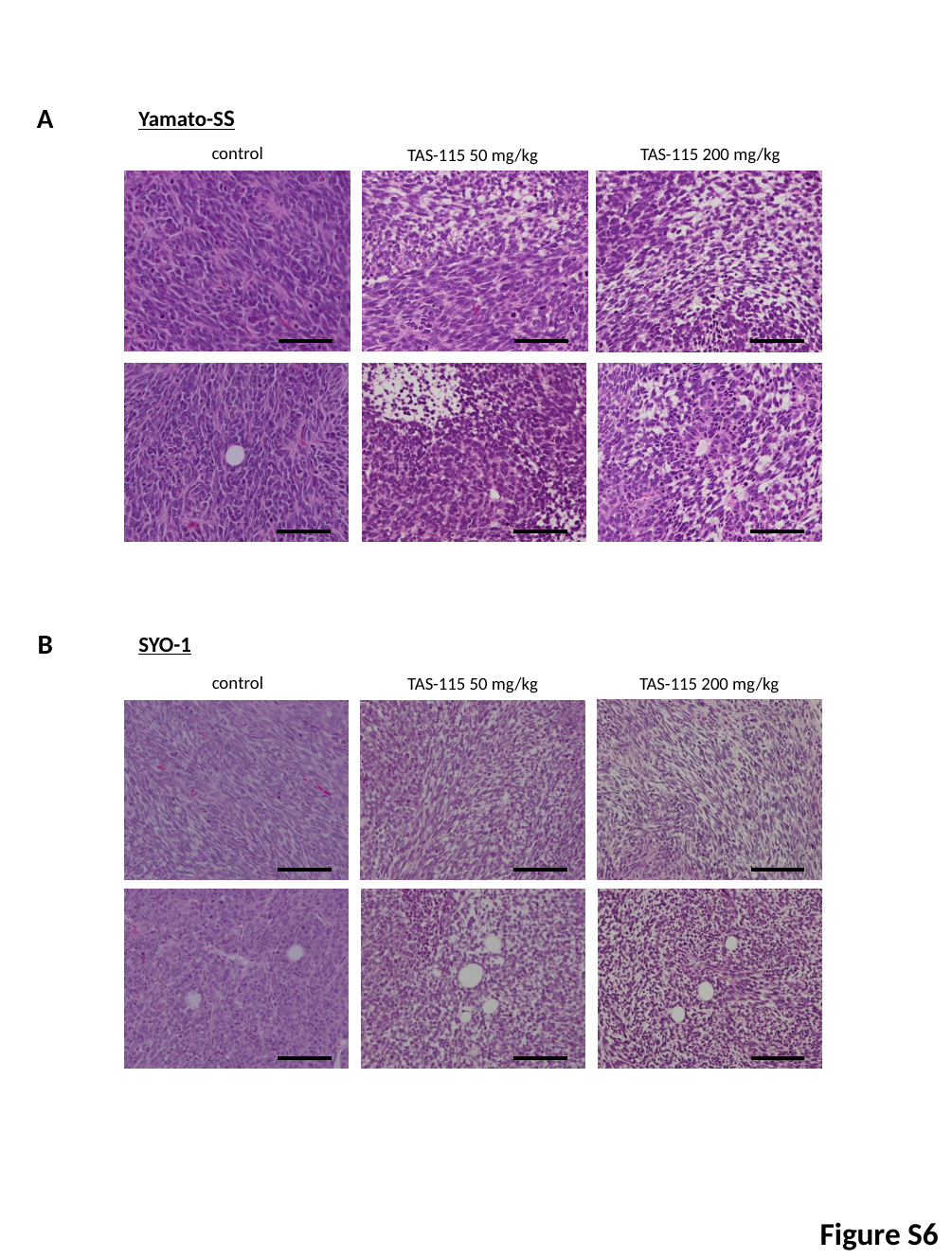

A
Yamato-SS
control
TAS-115 200 mg/kg
TAS-115 50 mg/kg
B
SYO-1
control
TAS-115 200 mg/kg
TAS-115 50 mg/kg
Figure S6

Supplement: Supplementary file 6 — (A) Light microscopic findings of Yamato-SS xenograft tumours for each treatment group (× 200). Scale bars, 100 μm. The tumours had both spindle cell components (upper panels) and epithelial cell components (lower panels). (B) Light microscopic findings of SYO-1 xenograft tumours for each treatment group (× 200). Scale bars, 100 μm. The tumours had both spindle cell components (upper panels) and epithelial cell components (lower panels). (PPTX 7168 kb) [file 12885_2017_3324_MOESM6_ESM.pptx]
