# Supplementary material for: Therapeutic potential of TAS-115 via c-MET and PDGFRα signal inhibition for synovial sarcoma
Source: BMC Cancer. 2017 May 16;17:334. doi: 10.1186/s12885-017-3324-3 (PMC5434537; doi:10.1186/s12885-017-3324-3)
Supplement: Supplementary file 7 — (A) Immunohistological staining of anti-CD31 antibody in Yamato-SS xenograft tumours for each treatment group (× 200). Scale bars, 100 μm. (B) Microvascular density (MVD) of Yamato-SS xenograft tumours. Bars represent the SD. * p < 0.05. (C) Immunohistological staining of anti-CD31 antibody in SYO-1 xenograft tumours for each treatment group (× 200). Scale bars, 100 μm. (D) Microvascular density (MVD) of SYO-1 xenograft tumours. Bars represent the SD. * p < 0.05. (PPTX 2778 kb) [file 12885_2017_3324_MOESM7_ESM.pptx]

## Slide 1
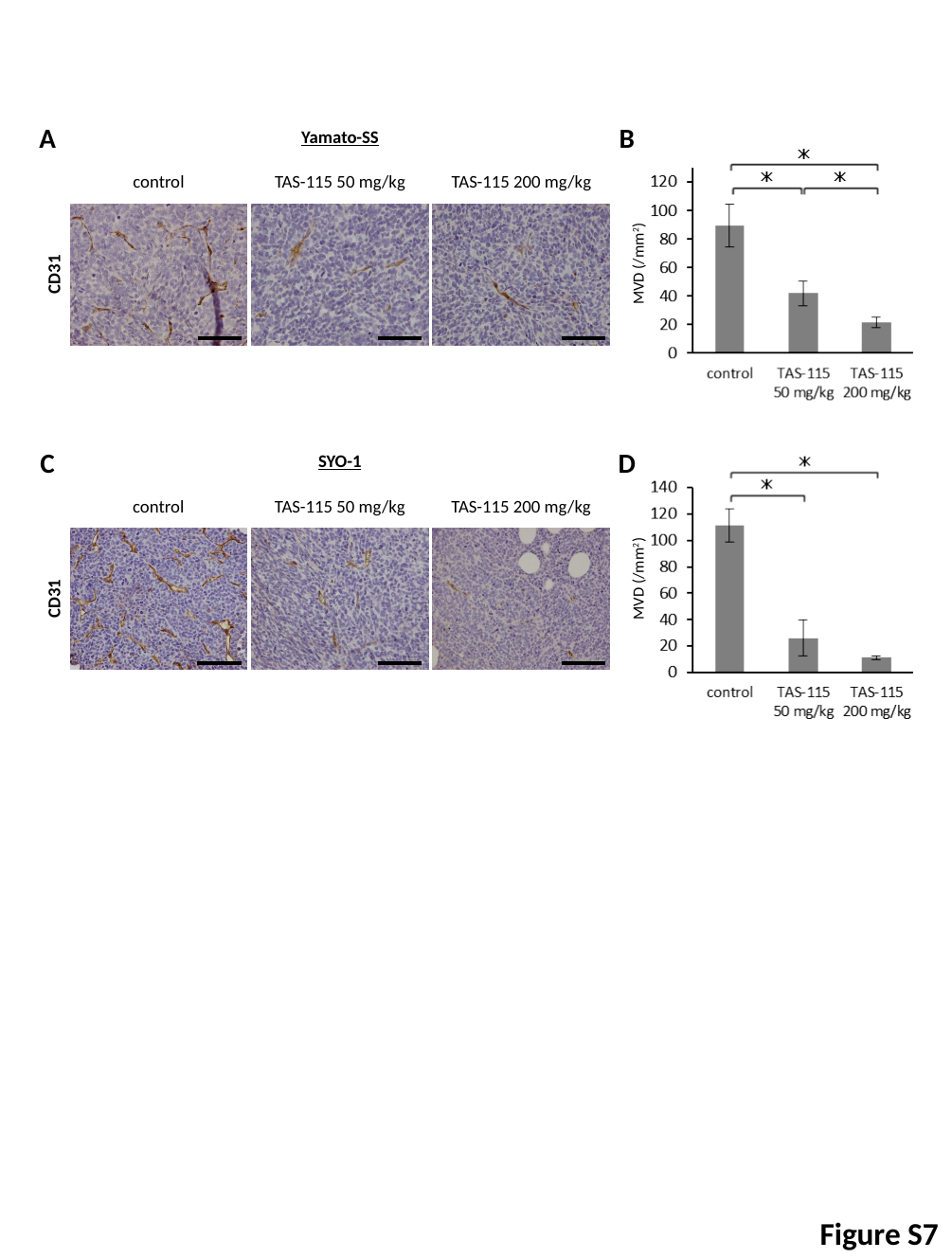

A
B
Yamato-SS
control
TAS-115 50 mg/kg
TAS-115 200 mg/kg
MVD (/mm2)
CD31
C
D
SYO-1
control
TAS-115 50 mg/kg
TAS-115 200 mg/kg
MVD (/mm2)
CD31
Figure S7
