# Supplementary material for: Therapeutic potential of TAS-115 via c-MET and PDGFRα signal inhibition for synovial sarcoma
Source: BMC Cancer. 2017 May 16;17:334. doi: 10.1186/s12885-017-3324-3 (PMC5434537; doi:10.1186/s12885-017-3324-3)
Supplement: Supplementary file 8 — Supplementary methods. (DOCX 16 kb) [file 12885_2017_3324_MOESM8_ESM.docx]

**Supplementary methods**

*Antibodies*

Antibodies against cytokeratin (AE1/AE3) (M3515; Dako), vimentin (ab17600; Abcam, Cambridge, UK) and CD31 (PECAM-1) (#77699, Cell Signaling Technology, Inc.) were used at a concentration of 1:100 for immunohistochemistry.

*Measurement of microvascular density*

Pictures were taken of 3 fields/section of each specimen of each treatment group in the best-stained tumour area (× 200). The microvascular density (MVD, number of vessels/mm^2^) was calculated using the equation given below:

MVD = (total number of vessels in 3 visual fields)/(area of one visual field × 3)
