# Supplementary material for: Therapeutic potential of TAS-115 via c-MET and PDGFRα signal inhibition for synovial sarcoma
Source: BMC Cancer. 2017 May 16;17:334. doi: 10.1186/s12885-017-3324-3 (PMC5434537; doi:10.1186/s12885-017-3324-3)
Supplement: Supplementary file 9 — Immunohistological staining of anti-vimentin and anti-cytokeratin (AE1/AE3) antibodies in SYO-1 xenograft tumours for each treatment group (× 400). Vimentin was diffusely present in both spindle and epithelial components, whereas epithelial cells expressed cytokeratin (AE1/AE3) relatively higher than spindle tumour cells. Scale bars, 100 μm. (PPTX 7779 kb) [file 12885_2017_3324_MOESM9_ESM.pptx]

## Slide 1
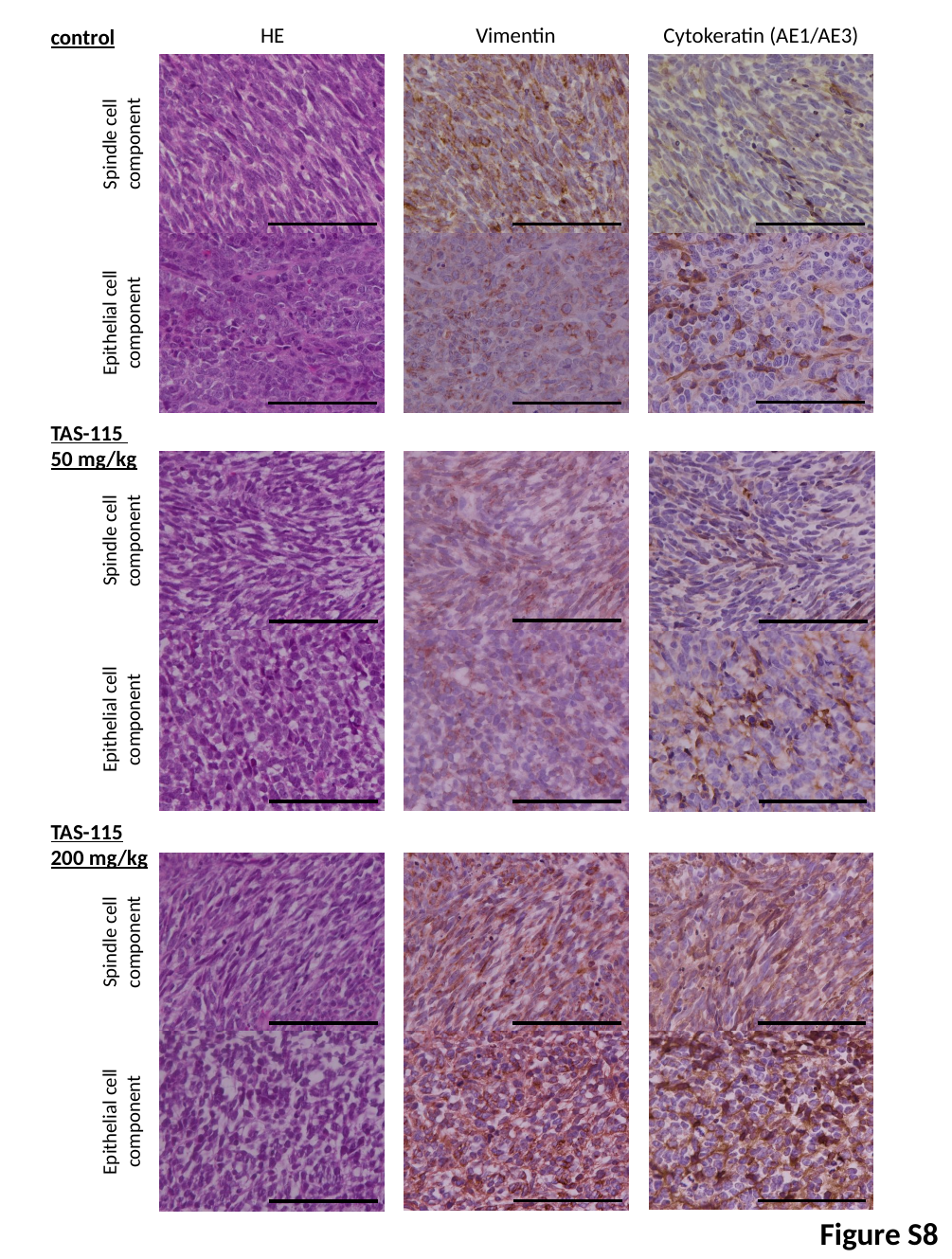

HE
Vimentin
Cytokeratin (AE1/AE3)
control
Spindle cell component
Epithelial cell component
TAS-115
50 mg/kg
Spindle cell component
Epithelial cell component
TAS-115
200 mg/kg
Spindle cell component
Epithelial cell component
Figure S8
